# Supplementary figures and images for: Applications of minimally invasive multimodal telemetry for continuous monitoring of brain function and intracranial pressure in macaques with acute viral encephalitis
Source: PLoS One. 2020 Jun 25;15(6):e0232381. doi: 10.1371/journal.pone.0232381 (PMC7316240; doi:10.1371/journal.pone.0232381)

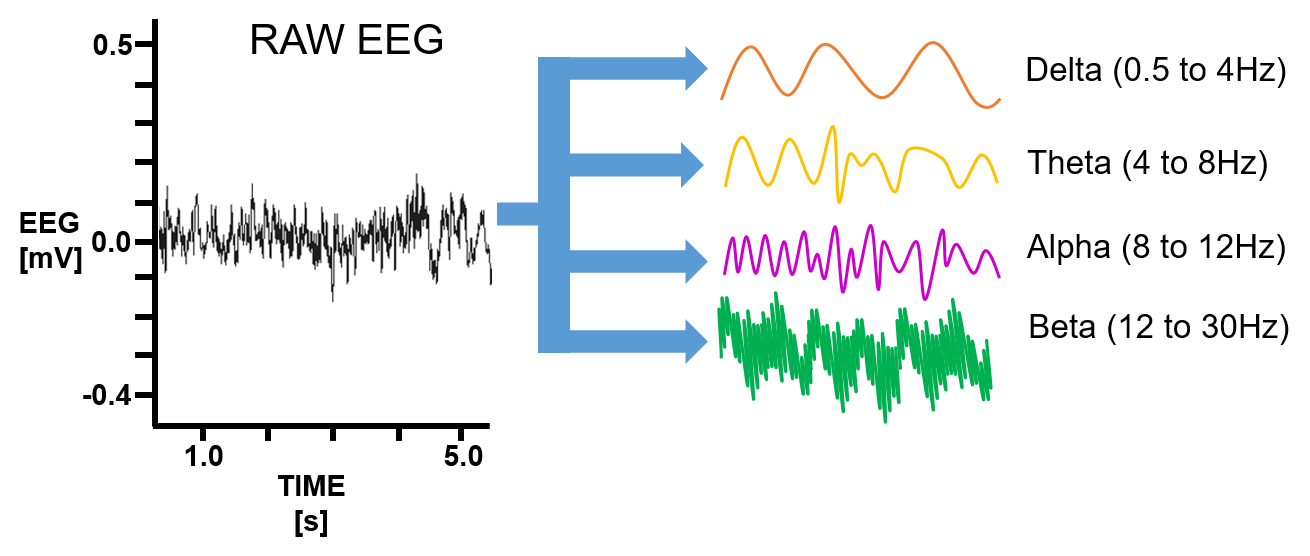

Supplement: S1 Fig — Raw data collected from the dedicated telemetry computer equipped with Ponemah software package provides a real-time, continuous display of EEG data, y-axis in units of mV. These data are downloaded to the NeuroScore software package and exported in .edf file extension format for analysis in MATLAB for frequency decomposition and reconstitution into delta, theta, alpha, and beta time series traces. (TIF) [file pone.0232381.s001.tif]
