# Supplementary material for: Applications of minimally invasive multimodal telemetry for continuous monitoring of brain function and intracranial pressure in macaques with acute viral encephalitis
Source: PLoS One. 2020 Jun 25;15(6):e0232381. doi: 10.1371/journal.pone.0232381 (PMC7316240; doi:10.1371/journal.pone.0232381)
Supplement: Attachment — Submitted filename: ResponseToReviewers.pdf [file pone.0232381.s005.pdf]

Dear Esteemed Reviewers and Editor:

Thank you for your comments and insights regarding our manuscript and for your time and consideration. Our responses are entered below, point-by-point, in relation to the comments received.

Journal Comments.

1. *Please ensure that your manuscript meets PLOS ONE's style requirements, including those for file naming. The PLOS ONE style templates can be found at https://journals.plos.org/plosone/s/file?id=wjVg/PLOSOne_formatting_sample_main_body.pdf and https://journals.plos.org/plosone/s/file?id=ba62/PLOSOne_formatting_sample_title_authors_affiliations.pdf*

   The manuscript was checked for style and author affiliation. There was an oversight during the initial submission:

   - Dr. Douglas S. Reed and Dr. Tobias Teichert are co-equal senior authors, and the manuscript has been changed to reflect this.

2. *We note that you have stated that you will provide repository information for your data at acceptance. Should your manuscript be accepted for publication, we will hold it until you provide the relevant accession numbers or DOIs necessary to access your data. If you wish to make changes to your Data Availability statement, please describe these changes in your cover letter and we will update your Data Availability statement to reflect the information you provide.*

   Code will be made available on GitHub, and data to be made available on request due to large file size.

3. *In order to comply with PLOS ONE's guidelines for non-human primate experiments (http://journals.plos.org/plosone/s/submission-guidelines#loc-non-human-primates), please indicate how often animal care staff monitored the health and well-being of the animals and specify the disposition of animals at the end of the study (e.g. euthanasia, returned to home colony, etc.). If animals were euthanized following the study, please provide the method of sacrifice.*

   Revisions made as requested (page 8 of revised manuscript, first paragraph from the top); method of sacrifice consisted of anesthetized induction of respiratory arrest.

__**Reviewer #1.**__

We thank the reviewer for their comments, and have formulated our responses enumerated in order of their appearance.

1. Line 104-105: Rewording of the sentence is recommended to improve clarity. For example, it may be revised to "However, these studies have technical limitations that may affect inter-rater reliability and reproducibility."

Revision made as requested; (page 4 of revised manuscript, first paragraph from the top). The paragraph in question now reads:

> *"While these and other studies have provided important insights, they were also constrained by technical limitations that could impact inter-rater reliability and reproducibility. In particular, determining the onset and resolution of encephalitis often depends on subjective and intermittent veterinary staff observations, rather than continuous and objective measurements of brain-specific parameters that can capture and quantify the complex and multi-modal manifestation of encephalitic disease…"*

2. In the introduction, it is not clear why continuous ICP monitoring would be important in alphavirus disease model. There are other methods that can be used to monitor a disease state "continuously and objectively" other than described brain monitoring devices, such as continuous EKG/telemetry and pulse oximeter for vitals. Thus, this reviewer recommends emphasizing the importance of continuous monitoring of brain-specific parameters based on a typical clinical course in humans in the introduction. For example, previous case reports of human EEEV included diffuse brain swelling as radiographic findings (N Engl J Med 1997; 336:1867-1874) and increased ICPs (Neurocrit Care. 2013 Aug;19(1):111-5, Emerg Infect Dis. 2016 Dec; 22(12): 2216–2217.). Particularly, rise in ICP and brain swelling can be a delayed finding that is observed a few days into the disease course, which argues for the need for continuous monitoring so that a timely treatment can be initiated. These are briefly mentioned in the later section under ICP monitor data, which may be introduced earlier to help readers follow the logic.

We thank the reviewer for the provision of relevant references which were included in the current version of the manuscript. Furthermore, we have now stressed the importance of continuous monitoring of brain-specific parameters earlier in the introduction. As suggested, we highlight the importance of continuous monitoring for initiating timely treatment in the clinical setting, and in our specific case, to evaluate the efficacy of medical countermeasures. We have revised the manuscript for clarity (page 5 of revised manuscript, first paragraph from the top); the paragraph now reads:

> *"…Literature from human clinical cases have demonstrated the appearance of abnormal EEG alongside radiography indicative of brain swelling [18]. Similarly, brain swelling and the putative rise in ICP are observed several days after the onset of alphavirus encephalitic disease in humans – continuous monitoring can thereby help to signal the need for timely intervention [19, 20]. More importantly, in the context of this animal*

model, continuous EEG/ICP can help to assess the safety and efficacy of medical countermeasures."

3. Similarly, the need for continuous EEG monitoring and in particular circadian index is unclear. Most clinicians use EEG to detect seizures even though it can also provide information about integrity of circadian rhythms. Is there evidence in alphavirus infection (or other similar diseases) that loss of circadian variation in EEG frequencies heralds or correlates with onset of important clinical changes (e.g. seizure, brain swelling)? Why this particular focus is important needs further explanation.

The loss of circadian variation in our EEG data co-occurs with clinical changes such as fever onset in EEEV and the second febrile peak of VEEV; this was a corroborative post hoc finding that reinforces similar findings in other animal models, mostly in the context of influenza in the murine model and the onset of fever in response to virus infection (Davis CJ, et al. 2017; Chen L, et al. 2004; Alt JA, et al. 2003. References listed at the end of response):

A human analogue exists in the detection of changes in EEG in individuals chronically infected with HIV (Darko DF, et al. 1995). The loss of circadian variation is likely caused by the effect of cytokines such as IL-6 on the biomolecular clock. We hypothesize that it may serve as a surrogate marker of the inflammatory response. In-depth overviews of these findings are currently being prepared for separate manuscripts detailing the pathology of EEEV and VEEV. Although seizure frequency is undoubtedly related to increases in cytokines or acute phase reactants (Vezzani A, et al. 2008).

The biomolecular milieu that underlies a condition such as brain swelling in viral encephalitis also pertains to dys/regulation of the circadian index. This is particularly important for research purposes, especially with regards to the evaluation of pathological changes in response to infection on long-term timescales. These are some of the same cytokines and acute phase reactants implicated in disrupting circadian EEG patterns (Cavadini G, et al. 2007; Majde JA, et al. 2005; Opp MR, et al. 1992; Yoshida H, et al. 2004).

This rationale has been included in an expansion of the introduction (page 5 of revised manuscript, first paragraph from the top). The paragraph now reads:

"…of relevant physiological measures [3, 12-14]. The loss of circadian variation is likely caused by the effects of cytokines such as IL-6 on the biomolecular clock, and as such, circadian rhythms can serve as a surrogate marker of the inflammatory response. Although other neurological events detectable by EEG such as seizures are undoubtedly related to increases in cytokines or acute phase reactants, the biomolecular milieu that underlies a condition such as brain swelling also pertains to disruption of the circadian rhythm. This is particularly important for alphavirus research goals, especially with regard to the evaluation of pathological changes in response to infection on long-term timescales [18-21]. Finally, we define…"

We have also incorporated a more detailed discussion of this aspect of the circadian index into the discussion section at the end of the EEG section (page 24 and 25 of revised manuscript, last and first paragraphs from the top, respectively). The paragraph now reads:

"Multiple reports in the literature comment on the loss of circadian variability in various segments of the EEG frequency spectrum during viral infection, with various empirical associations outlined that implicate both systemic and neurogenic origins of circadian disruption [56-63]. Acute phase reactants such as tumor necrosis factor (TNF-α) have

been tied to slow-wave EEG activity, including the delta power band; TNF-α is thought to directly or indirectly regulate somnolence [63]. Endogenous fluctuations of inflammatory or immune species in response to infection may thereby produce changes detectable by EEG. Our findings hint at an inflammatory neurogenic origin of disrupted circadian rhythmicity. For instance, the first febrile peak in VEEV is associated with virus dissemination through the lymphatic system, while the second febrile period is proposed to be associated with infection of the CNS [12, 40]. Circadian disruptions of EEG in VEEV infection occur largely during the second febrile peak, suggesting that infection of the brain parenchyma and/or the subsequent immune response cascade precipitates the disruption. Holistically, the choice to present findings pertaining to the circadian index arose out of the practical consideration to illustrate the longitudinal nature of brain pathology in the context of alphavirus encephalitis. This consideration leverages the continuous nature of circadian index construction over the assessment of seizures, which did not occur in all macaques with alphavirus encephalitis and which presented nonuniformly and episodically. Evaluating seizure activity in cynomolgus macaques would not be useful in assessing efficacy of medical countermeasures against alphavirus encephalitis. With respect to the infectious origin of alphavirus encephalitis, the biomolecular milieu that facilitates encephalitis also affects, as described above, the regulation and dysregulation of the circadian variation of EEG, captured by the circadian index. Our findings suggest that the assessment of the circadian index can provide a valuable research tool for the determination of the onset, resolution, and/or mitigation of disease in the context of the evaluation of medical countermeasures against acute viral encephalitides."

Davis CJ, et al. Neurobiology of sleep and circadian rhythms. 2017;3:1-9.
Chen L, et al. Journal of applied physiology. 2004;97(1):17-28.
Alt JA, et al. Journal of applied physiology. 2003 Aug;95(2):460-8.
Darko DF, et al. Proceedings of the National Academy of Sciences. 1995;92(26):12080-4.
Vezzani A, et al. Brain, behavior, and immunity. 2008 Aug 1;22(6):797-803.
Cavadini G, et al. Proc Natl Acad Sci. 2007;104(31):12843-8.
Majde JA, Krueger JM. J Allergy Clin Immunol. 2005;116(6):1188-98.
Opp MR, Kapás L, Toth LA. Proc Soc for Exp Biol and Med. 1992;201(1):16-27.
Yoshida H, et al. Brain Research. 2004;1009(1):129-36.

4. It is described that infected macaques exhibit seizures. While EEG can be used for power frequency analysis and circadian index calculation, the most common clinical use of EEG is to detect seizures. Please describe how seizures were defined (e.g. behaviorally on human observation, per EEG findings, or combination), and how each seizure episode affected subsequent EEG findings (e.g. post-ictal slowing/increase in delta power), and whether any anti-seizure medication was attempted. Detailed description of seizures would also be important, regarding semiology (unilateral limb shaking, facial twitching, generalized shaking, staring, drooling, incontinence, etc.), duration, and frequency over the course of disease after viral infection. Seizures in human transiently increase ICP, and secondary cascade can lead to accelerated brain swelling and fatal rise in ICP (similar to what is described here). Therefore, the relative timing of observed seizure in ICP tracing is a clinically relevant information. As the authors mention in the limitation, a single lead EEG will have limited use in detecting

seizures, especially focal seizures that have been described in human EEEV cases. However, if the field caused by the seizure is large enough, it likely affects the frequency bands of the single-lead EEG.

Seizure detection was an ancillary goal of the use of EEG in this study, but we agree with the reviewer that the clinical use of EEG in humans mainly pertains to the detection of seizures.

In our study, seizures were detected either through incidental observation or in-depth review of video recordings. Due to the time-intensive nature of the process, in-depth videos were compiled only for a subset of macaques. Seizure activity was observed incidentally in 50% of EEEV-infected macaques. In contrast, macaques infected with VEEV did not display seizure activity.

Observed seizures typically comprised tonic-clonic seizures with generalized shaking and immobility for approximately 3-5 seconds' duration and a post-ictal period of approximately 5-10 seconds. These seizures were observed with greatest frequency at 4-6 DPE in EEEV-infected macaques.

As these macaques were employed in a natural history study, anti-seizure medication was not used to prevent interference with the disease course and more importantly to prevent interference with EEG signal output (pages 9 and 10 of revised manuscript, last and first paragraphs from the top, respectively).

> *"Subject welfare was assessed on a daily basis, at least once per day by a series of ordinal scales for: neurology, activity, and temperature [3, 12, 13]. All scoring scales ran from 1-6. The neurology scale accounted for signs of neurological disease such as involuntary tremor, gait imbalance, nystagmus, head pressing, seizures, and coma. Seizures were defined primarily by incidental human observation on welfare assessment and secondarily by visual review of recorded video data. The activity scale accounted for posture, facial expressions, responses to stimuli, and interactions with observers. Finally, the temperature scale accounted for core temperature set at standard deviations above or below mean core temperature to detect fever or hypothermia. The cumulative score determined by summing all three scales' scores was used to determine whether a macaque required more frequent observation or was at risk of imminent death which triggered the humane study endpoint. No analgesia or anti-seizure medications were employed to minimize potential impact on EEG and ICP data collection."*

We were not able to identify reliable pre- or post-ictal EEG corelates. As pointed out by the reviewer, this was likely due to the poor spatial resolution of the two-lead EEG system. Therefore, we described the results only in passing and only as pertinent to the EEEV disease course in a qualitative manner.

To date, we have not tested whether ICP increases in the post ictal period – only one ICP-monitored macaque in this study cohort manifested seizures detected by in-depth video review.

We have revised the manuscript to address the reviewer's points. The paragraph in question (page 27 of revised manuscript, second paragraph from the top) now reads:

> *"The augmentation of this well-established macaque model holds utility towards the development of improved vaccines. The macaques that exhibited courses of EEEV or VEEV disease displayed abnormal EEG and ICP measurements. Observed seizures occurred only in EEEV-infected macaques and typically comprised tonic-clonic seizures with generalized shaking and immobility for approximately 3-5 seconds' duration and a*

post-ictal period of approximately 5-10 seconds. These seizures were observed with greatest frequency at 4-6 DPE in EEEV-infected macaques. Seizures tended to occur on days with abnormal EEG. However, it was not possible to reliably identify seizure activity in the EEG. This may be due in part to the spatial limitations of our two-lead EEG system."

5. Line 446-448: This reviewer disagrees with the statement that monitoring of ICP serves an intuitive and proximal indicator of the response to infection within the brain, especially when an extradural ICP monitor is used. ICP reflects the pressure within the system (in human, ICP tracing commonly reflects pressure transmitted through the CSF space with the use of endoventricular drain), and increased ICP is more commonly a delayed and indirect finding of brain injury (which ultimately leads to inflammation and swelling) than a proximal measure of brain response. In addition, focal abnormalities in the deep brain structures such as thalami and lenticular nuclei have been reported in EEEV encephalitis, which potentially affect the venous drainage and CSF circulation, resulting in secondary rise in ICP that may be picked up by extradural ICP monitor once enough pressure builds up. A revision of the statement is recommended to avoid making controversial points.

We agree with the concerns of the reviewer. This statement, as well as the one that gave rise to comment 6 below, is best understood in contrast to the dependent measures collected in earlier experiments, i.e. temperature and electrocardiography. Relative to these measures, both EEG and ICP are substantially more proximal to the affected organ, i.e. the brain. But we certainly agree with the comment of the reviewer and revisions were made as requested (page 19 and 20 of revised manuscript, last and first paragraphs from the top, respectively); the paragraph in question now reads:

"Increased ICP during alphavirus encephalitis has been reported in humans [41-45]. Increases of ICP are typically a delayed and indirect result of brain injury, which ultimately leads to inflammation and swelling; in addition, inflammation and swelling of deep brain structures can also affect venous drainage and CSF circulation, thus leading to a secondary rise in ICP. To measure ICP in the macaques, we inserted a standard blood-pressure sensor in between the dura and the skull. Because blood pressure is much higher than intracranial pressure…"

6. Line 542-544: This reviewer disagrees with the statement that EEG is a direct measure of brain function. EEG is a electrophysiological marker of overall brain activity and does not measure any specific function of the brain. A revision is recommended, such as "The continuous EEG monitoring over multiple days in mock-infected animals allowed us to characterize normal variations across time of the day, reflecting normally present biological rhythms in different frequency ranges of brain activity."

The reviewer makes a good point. EEG activity is not a measure of the firing of individual neurons. Rather, it is driven by post-synaptic potentials that are synchronized across a large number of neurons. In specific experimental settings, high-density EEG can indeed be used to study highly specific aspects of brain function such as the detection of an event that violates a

previously established expectation. But as the reviewer points out, our observational setup, in combination with only two EEG leads does not provide this level of functional specificity. Hence, our original statement was not optimally worded. The beginning of the paragraph was revised as requested (page 23 and 24 of revised manuscript, last and first paragraphs from the top, respectively). It now reads:

> *"EEG is a biopotential signal that arises from synchronous post-synaptic potentials in large groups of neurons. Key aspects of EEG are conserved across many species and thus applicable to macaques and humans. The continuous long-term monitoring in mock-infected animals allowed us to characterize normal variations across the time of day, reflecting normally present biological rhythms in different frequency ranges of brain activity. This data established a baseline against which to evaluate pathological brain function caused by infection with an encephalitic alphavirus…"*

Reviewer #2.

We thank the reviewer for their comments, and have formulated our responses enumerated in order of their appearance.

1) Line 90-92. Rhesus Macaques were utilized in the study referenced. Also, the first study of EEEV in NHP was the reference below.
E. Weston Hurst. Infection of the rhesus monkey (Macaca mulatta) and the guinea-pig with the virus of equine encephalomyelitis. The Journal of Pathology and Bacteriology. 1936.

Reference replaced with relevant references (page 3 of revised manuscript, last paragraph from the top). Suggested reference incorporated in response to the comment below.

2) Line 531 the 1st EEEV studies were in Rhesus macaques. Please see the reference above. Also reference 48 is not correct, as there is no mention of NHP in the study.

We acknowledge that the paper by Beck, et al. did not mention NHP in the study; the citation was intended to provide a reference to one of the seminal papers regarding the discovery of VEEV. Reference removed, and revision made as requested (page 23 of revised manuscript, second paragraph from the top). The paragraph now reads:

> "Nonhuman primates, including the cynomolgus macaque (Macaca fascicularis) have served as model organisms for equine encephalitis viruses since the discovery of these viruses in the 1930s [52, 53]."

3) Line 74 "Alphaviruses" is not a genus. The genus Alphavirus is in the Togaviridae family.

Revision made as requested (page 3 of revised manuscript, first paragraph from the top). The sentence now reads:

> "The genus Alphavirus of the family Togaviridae composes a group of linear, non-segmented positive-sense single-stranded RNA arboviruses found in many regions of the world."

4) Line 75 "New World". Remove quotation sign.

Revision made as requested (page 3 of revised manuscript, first paragraph from the top).

5) Line 83, remove "," after natural.

Revision made as requested (page 3 of revised manuscript, first paragraph from the top).

6) Line 86, please reword sentence is awkward.

Revision made as requested (page 3 of revised manuscript, last paragraph from the top). The sentence now reads:

> "Aerosol transmission of VEEV and EEEV does not comprise a natural route of transmission; because of disease severity and the lack of natural infections, the evaluation of safety and efficacy for potential medical countermeasures must proceed in animals in accordance with the FDA's 'Animal Rule.'"
